# Detection of clinically significant prostate cancer with PI-RADS v2 scores, PSA density, and ADC values in regions with and without mpMRI visible lesions

**DOI:** 10.1590/S1677-5538.IBJU.2018.0768

**Published:** 2019-09-02

**Authors:** Antonio C. Westphalen, Farhad Fazel, Hao Nguyen, Miguel Cabarrus, Katryana Hanley-Knutson, Katsuto Shinohara, Peter R. Carroll

**Affiliations:** 1 Department of Radiology and Biomedical Imaging, University of California, San Francisco, CA, USA;; 2 Department of Urology, University of California, San Francisco, CA, USA;; 3 Helen Diller Family Comprehensive Cancer Center, University of California, San Francisco, CA, USA

**Keywords:** Radiology, Prostate, Magnetic Resonance Imaging

## Abstract

**Purpose:**

To determine if PSAD, PSADtz, and ADC values improve the accuracy of PI-RADS v2 and identify men whose concurrent systematic biopsy detects clinically significant cancer on areas without mpMRI visible lesions.

**Materials and methods:**

*S*ingle reference-center, cross-sectional, retrospective study of consecutive men with suspected or known low to intermediate-risk prostate cancer who underwent 3T mpMRI and TRUS-MRI fusion biopsy from 07/15/2014 to 02/17/2018. Cluster-corrected logistic regression analyses were utilized to predict clinically significant prostate cancer (Gleason score ≥3+4) at targeted mpMRI lesions and on systematic biopsy.

**Results:**

538 men (median age=66 years, median PSA=7.0ng/mL) with 780mpMRI lesions were included. Clinically significant disease was diagnosed in 371 men. PI-RADS v2 scores of 3, 4, and 5 were clinically significant cancer in 8.0% (16/201), 22.8% (90/395), and 59.2% (109/184). ADC values, PSAD, and PI-RADS v2 scores were independent predictors of clinically significant cancer in targeted lesions (OR 2.25-8.78; P values <0.05; AUROC 0.84, 95% CI 0.81-0.87). Increases in PSAD were also associated with upgrade on systematic biopsy (OR 2.39-2.48; P values <0.05; AUROC 0.69, 95% CI 0.64-0.73).

**Conclusions:**

ADC values and PSAD improve characterization of PI-RADS v2 score 4 or 5 lesions. Upgraded on systematic biopsy is slightly more likely with PSAD ≥0.15 and multiple small PI-RADS v2 score 3 or 4 lesions.

## INTRODUCTION

The Prostate Imaging Reporting and Data System (PI-RADS) (1) has standardized the diagnosis of prostate cancer using multiparametric magnetic resonance imaging (mpMRI). Improved characterization of cancer identified on mpMRI may be achieved with the incorporation of prostate-specific antigen density (PSAD) and apparent diffusion coefficient (ADC) values to the PI-RADS v2 guidelines (2, 3). Older studies in the urology literature support the use of transition zone adjusted PSAD (PSADtz), calculated from prostate volumes estimated using transrectal ultrasound (TRUS), to improve the prediction of cancer grade in men with low and intermediate total serum PSA range (4, 5), but little data has been produced since the advent of mpMRI.

The interpretation of continuous variables, especially those with a large number of possible values, e.g. PSAD, PSADtz, and ADC values, is not simple. An approach that simplifies the use of such results in clinical practice is stratification into categories that represent different risks of an outcome. This strategy is often utilized; for example, PI-RADS states that lesions with ADC values below 750 to 900 x 10^-6^mm^2^/s are more likely to represent prostate cancer (1). And a PSAD of greater than 0.15 has been shown to be associated with a higher rate of Gleason ≥3+4 disease in patients with a positive mpMRI (2). Although the incorporation of these strategies has been suggested, further validation is required.

Concurrent systematic biopsies are performed in the vast majority of men who undergo TRUS-MRI fusion biopsy. This is because PI-RADS v2 scores do not adequately predict the identification of clinically significant prostate cancer in regions sampled on systematic biopsy that are negative on mpMRI (6, 7). For increased clarity, perhaps we could modify this paragraph slightly as suggested below.

PSAD, PSADtz, and ADC values may help to identify which men with visible lesions on mpMRI have a higher risk of having a clinically significant tumor detected on conventional systematic biopsy. If so, the combined procedure could be reserved for those men. Other patients could undergo targeted TRUS-MRI fusion biopsy only and avoid the unnecessary sampling.

Accordingly, the goals of this study are to determine if PSAD, PSADtz, and ADC values improve the accuracy of PI-RADS v2 and identify men whose concurrent systematic biopsy detects clinically significant cancer on areas without mpMRI visible lesions.

## MATERIALS AND METHODS

This is a retrospective single institution study, approved by the institutional review board, and compliant with the United States Health Insurance Portability and Accountability Act of 1996. Informed consent was waived.

### Population

All consecutive men with suspected or known low to intermediate-grade prostate cancer who underwent mpMRI from 07/15/2014 to 02/17/2018, followed by a TRUS-MRI fusion biopsy, were eligible.

### Inclusion criteria

- If known cancer, Gleason scores 3+3 or 3+4
- 3-Tesla endorectal mpMRI
- PI-RADS v2 scores 3 to 5

### Exclusion criteria

- Men without focal abnormalities on mpMRI (PI-RADS v2 scores 1 or 2)
- Patients submitted to TRUS-guided systemic biopsy alone, without MRI-fusion biopsy
- Non-retrievable clinical, imaging, or pathological data
- Artifact precluding imaging interpretation

Men without focal abnormalities on mpMRI. i.e. those who were assigned a PI-RADS scores 1 or 2, were not included in this study because these patients undergo systematic TRUS-guided biopsy, rather than TRUS-MRI fusion biopsy.

### Data collection

Patients were identified through a search of imaging reports using Nuance mPower Clinical Analytics® (Nuance Communications, Inc. Burlington, Massachusetts). Our standardized report allowed us to find all scans done within the time frame using the key word “PI-RADS v2”. Additional data was obtained from our electronic radiology and medical records. Two authors collected all data. A third author performed a QA review of a random sample of the data.

The following data were acquired: age, race/ethnicity, family history of prostate cancer, baseline PSA, presence of palpable nodule, history and results of previous biopsy (none, benign, positive, and highest Gleason score), mpMRI prostate volume, mpMRI volume of the transition zone, number of lesions on mpMRI, lesion mpMRI characteristics (peripheral or transition zone, PI-RADS v2 score, three-plane diameters, volume, mean ADC value), lesion Gleason scores obtained with TRUS-MRI fusion biopsy, and highest Gleason score on systematic biopsy. PSAD and PSADtz were calculated dividing the baseline total serum PSA value by the prostate volume and transition zone volume, respectively (4, 5).

### MRI protocol

Images were acquired on a 3-Tesla magnet (Discovery^TM^ MR750 or Discovery^TM^ MR750w GEM (GE Healthcare LLC, Arlington Heights, IL) using an endorectal coil (MEDRAD^®^ Prostate eCoil, Bayer HealthCare LLC. Whippany, NJ). The protocol followed the PI-RADS v2 guidelines and included high-resolution T2-weighted images, high b-value diffusion-weighted images, and dynamic contrast-enhanced images (1). Details are provided in Appendix 1.

### Interpretation

**Figure 1:**
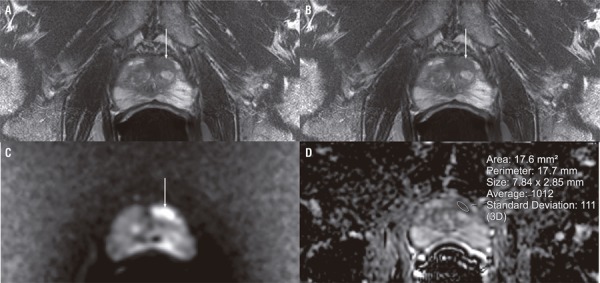
75-year-old man with suspected prostate cancer. Total serum PSA=8.5ng/mL. Prostate volume=41.2cc. Transition zone volume=24.3cc. PSAD=0.21. PSADtz=0.35. Images demonstrate a 1.2cm PI-RADS v2 4 lesion in the left apex anterior transition zone (arrows). Mean ADC value=1012x10−6mm2/s (region-of-interest on D). TRUS-MRI fusion biopsy diagnosed Gleason 3+4 prostate cancer. No other focus of high-grade prostate cancer was diagnosed on systematic biopsy. (A) T2-weighted image, (B) dynamic contrast enhanced (DCE) upslope parametric map, (C) diffusion-weighted image (DWI), (D) apparent diffusion coefficient (ADC) map.

Scans were interpreted by one of 13 board-certified, fellowship-trained, abdominal radiologists as part of clinical care. Approximately 75% of cases were interpreted by one of 5 radiologists, one of whom reviewed one third of the cases and the others approximately 10% each. Images were evaluated according to PI-RADS v2 and suspicious findings assigned a score 3 or higher (1). Up to 4 lesions were identified per patient. Mean ADC values were measured at a single slice depicting the most suspicious area of the lesion. Regions-of-interest were drawn to cover between 50% and 75% of the diameter of the lesion, as is customary at our institution (Figure-1). The gland and lesions were outline in DynaCAD for Prostate® (Invivo, Gainesville, FL, USA) for subsequent TRUS-MRI fusion biopsy.

### TRUS-MRI fusion biopsies

TRUS-MRI fusion biopsies were performed by subspecialized urologists as part of clinical care using UroNav Fusion Biopsy System® (Invivo, Gainesville, FL, USA) and 18-gauge needles. Based on the size of the target, one or two samples were taken from the center of the lesion and one or two cores from its periphery. This was followed by a 14-core extended-sextant systematic biopsy. Targeted and systematic biopsies were performed during the same session, by the same urologist. One of 4 urologists performed all procedures, but over 95% of these were done by two urologists with more than 2 years of experience with TRUS-MRI fusion biopsy.

### Histological analysis

Specimens were fixed on formalin and H&E stained; immunohistochemistry was performed when deemed necessary by the pathologist. Subspecialized genitourinary pathologists (experience ranging from 3 years to 18 years) interpreted the specimens using the International Society of Urological Pathology guidelines. Targeted and systematic samples were identified separately.

### Statistical analysis

Histopathology results were the standard of reference. According to the PI-RADS v2 guidelines (1), our outcome was clinically significant prostate cancer, defined as a Gleason score ≥3+4. The units of analyses were a) the individual mpMRI lesion and b), individual patient upgrade on systematic biopsy. Upgrade on systematic biopsy was defined as the identification of a tumor on systematic biopsy with a Gleason score of at least 3+4 and higher than the Gleason score of the targeted lesions.

Logistic regression analyses were utilized to predict clinically significant prostate cancer at targeted mpMRI lesions and to predict upgrade. Analyses were cluster-corrected to account for the possibility of more than one lesion per patient. Forward and backward selection were utilized to identify variables for inclusion in the multivariate models. ADC values were categorized into four groups, high (above 1100×10^−6^mm^2^/s), mildly low (between 1100 and 900×10^−6^mm^2^/s), moderately low (between 900 and 750×10^−6^mm^2^/s), and markedly low (below 750×10^−6^mm^2^/s). These cutoff values were chosen based on the suggestions of PIRADS and previous publications (1, 8). PSAD was similarly stratified in four categories: low (less than 0.15ng/mL/mL), mildly high (between 0.15 and 0.20ng/mL/mL), moderately high (between 0.20 and 0.25ng/mL/mL), and markedly high (above 0.25ng/mL/mL). We made this option because 0.15ng/mL/mL is a commonly used threshold in urology, and followed this by 0.5 increments. The area under the receiver-operating characteristic (ROC) curves were estimated and bootstrapping used to calculate 95% confidence intervals. We tested the equality of ROC curves utilizing the roccomp routine. Analyses were performed using Stata 13.1® (StataCorp LP, College Station, TX, U.S.A.). All tests were two-tailed and a 5% level of confidence was considered significant.

## RESULTS

### Demographics

761 men were eligible to this study, but after applying the inclusion and exclusion criteria, 538 were included. 198 (36.8%) were biopsy-näive, 55 (10.2%) had a prior negative biopsy, and 285 (53.0%) were under active surveillance. The median age and baseline PSA were 66 years (IQR=61-70) and 7.0ng/mL (IQR=5.5-9.8). The median interval between mpMRI and biopsy was 57 days (IQR=27-112). Table-1 provides further detail.

**Table 1:** Baseline characteristics and imaging findings.

Age (years) *	66 (61-70)
Race/ethnicity **	
American indian or Alaska native	1 (0.2)
Asian	35 (6.5)
Black/african-american	19 (3.5)
Hispanic or latino	20 (3.7)
Native hawaiian or another pacific island	1 (0.2)
White/caucasian	428 (79.6)
Other	34 (6.3)
Family history of prostate cancer **	
Yes	136 (25.3)
No	402 (74.7)
Palpable nodule on DRE **	
Yes	58 (10.8)
No	480 (89.2)
Biopsy prior to MRI **	
None	198 (36.8)
Benign	55 (10.2)
3+3	234 (43.5)
3+4	51 (9.5)
Baseline PSA (ng/mL) *	7 (5.5-9.9)
Prostate volume (cm^3^) *	50.0 (37.0-74.0)
Transition zone volume (cm^3^) *	26.0 (14.5-47.8)
PSA density *	0.14 (0.10-0.21)
PSA density categorical **	
Low (≤ 0.15)	295 (54.8)
Mildly high (0.15-0.20)	82 (15.2)
Moderately high (0.20-0.25)	69 (12.8)
Markedly high (≥ 0.25)	92 (17.1)
Transition zone adjusted PSA density *	0.28 (0.16-0.51)
PI-RADS v2 scores (peripheral zone) **	
3	159 (25.4)
4	343 (54.9)
5	123 (19.7)
PI-RADS v2 scores (transition zone) **	
3	42 (27.1)
4	52 (33.6)
5	61 (39.4)
Number of lesions/patient **	
1	335 (62.3)
2	167 (31.0)
3	29 (5.4)
4	7 (1.3)
Lesion maximum diameter (cm) *	1.3 (0.9 to 1.7)
Lesion volume (cm^3^) *	0.32 (0.14 to 0.71)
ADC values (x 10^−6^ mm^2^/s) *	1004 (287.7)
ADC categorical **	
Very low (≤ 750)	114 (21.2)
Low (750-900)	131 (24.4)
High (900-1100)	120 (22.3)
Very high (≥ 1100)	168 (31.2)
Missing	5 (0.9)

**DRE** = digital rectal examination; **PSA** = prostate specific antigen; **MRI** = magnetic resonance imaging; **PI-RADS v2** = Prostate Imaging Reporting and Data System, version 2; **ADC** = apparent diffusion coefficient; * = median (interquartile range); ** = count (percentage)

### PSAD and Imaging Findings

Table-1 also displays the imaging findings, PSAD, and PSADtz of the sample. The median prostate and transition zone volumes were 50.0cm^3^ (IQR=37.0-74.0) and 26.0cm^3^ (IQR=14.5-47.8). 780 PI-RADS v2 score 3 to 5 lesions were identified on mpMRI; most were in the peripheral zone (625/780, 80.1%).

### Biopsy

Gleason score ≥3+4 was diagnosed in PI-RADS v2 scores of 3, 4, and 5 in 8.0% (16/201), 22.8% (90/395), and 59.2% (109/184) of lesions (Table-2).

**Table 2:** Biopsy results by PI-RADS v2 scores.

		ISUP Group (Gleason Score)						
		Benign	1 (3+3)	2 (3+4)	3 (4+3)	4 (8)	5 (9-10)	Total
PIRADS v2 Score	3	144	41	12	2	1	1	201
	4	190	115	70	13	5	2	395
	5	35	40	69	24	5	11	184

	**Total**	**370**	**196**	**150**	**39**	**11**	**14**	**780**

**ISUP** = International Society of Urological Pathology; **PI-RADS v2** = Prostate Imaging Reporting and Data System, version 2.

Clinically significant prostate cancer was diagnosed in 371 men (69.0%, 371/538). Targeted biopsy identified 38 of these men (10.2%, 38/371); systematic biopsy, 157 (42.3%, 157/371), and both approaches, 176 (47.4%, 176/371).

Of the 157 men with clinically significant disease detected only on systematic biopsy, 56 were biopsy-näive patients (35.7%; negative mpMRI targets=31; Gleason 3+3 on targets=25). Ten had prior negative biopsy (6.4%; negative mpMRI targets=8; Gleason 3+3 on targets=2). And 91 were men under active surveillance (58.0%; negative mpMRI targets=48; Gleason 3+3 on targets=43).

### Logistic Regression-Targeted mpMRI Lesions

**Figure 2:**
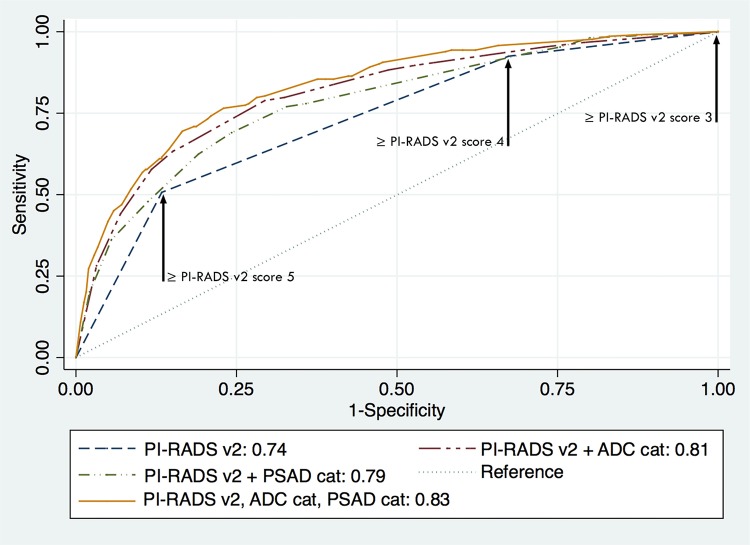
Prediction of clinically significant prostate cancer at targeted lesions, ROC curves. The greatest separation between the curve of the model that included only PI-RADS v2 scores and the other three models is seen in the segment that corresponds to PI-RADS v2 scores 4 and 5. The model that incorporated both categorized (cat) ADC values and PSAD provided better discrimination.

Only age, palpable nodule, PI-RADS v2 score, and categorical ADC were identified by both forward and backward selection models to be included in the multivariate models. Additionally, the multivariate models included PSAD, categorical PSAD, or PSADtz. Table-3 summarizes the results of these analyses, including the areas under the ROC curves, which varied from 74% to 84%. The areas under the ROC curves of all models were significantly higher than the area under the ROC of PI-RADS v2 alone (all P <0.001). The area under the ROC of PI-RADS v2 associated with PSAD was larger than the area of PI-RADS v2 and PSAD categorical (difference=1.87%, P <0.001), but no differences were seen when PSADtz was compared to PSAD (difference=0.84%, P=0.12) or to PSAD categorical (difference=1.03%, P=0.16). The area under the ROC curve of PI-RADS v2 plus ADC and PI-RADS plus ADC categorical were not significantly different (difference=1%, P=0.24). Figure-2 illustrates some of these results.

**Table 3:** Targeted lesion – logistic regression analyses.

	Odds Ratio	P	95% CI	
	
	PI-RADS v2 alone			
**PI-RADS v2**				
4	3.41	<0.001	1.92	6.08
5	16.80	<0.001	9.24	30.55

	AUROC = 0.74 (95% CI = 0.70-0.77)			

	PI-RADS v2 and ADC			

**PI-RADS v2**				
4	2.18	0.01	1.18	4.04
5	6.91	<0.001	3.69	12.93
ADC	0.996	<0.001	0.995	0.997

	AUROC = 0.82 (95% CI = 0.78-0.85)			
	
	PI-RADS v2 and ADC categories			

**PI-RADS v2**				
4	2.22	0.01	1.21	4.08
5	6.99	<0.001	3.75	13.05
**ADC categories**				
2	2.00	0.025	1.09	3.67
3	4.40	<0.001	2.49	7.79
4	10.59	<0.001	5.85	19.17

	AUROC = 0.81 (95% CI = 0.77-0.85)			
	
	PI-RADS v2 and PSAD categories			

**PI-RADS v2**				
4	3.47	<0.001	1.92	6.25
5	15.02	<0.001	8.14	27.73
**PSAD categories**				
2	2.59	<0.001	1.54	4.36
3	2.86	<0.001	1.65	4.98
4	4.59	<0.001	2.76	7.61
	AUROC = 0.79 (95% CI = 0.74-0.82)			
	PI-RADS v2 and PSAD			
**PI-RADS v2**				
4	3.47	<0.001	1.89	6.38
5	14.89	<0.001	7.88	28.12
PSAD	434.39	<0.001	52.47	3596.55

	AUROC = 0.80 (95% CI = 0.75-0.82)			
	
	PI-RADS v2 and PSADtz			

**PI-RADS v2**				
4	3.37	<0.001	1.80	6.31
5	15.18	<0.001	7.94	29.04
PSAD transition zone	3.44	<0.001	1.88	6.32
	AUROC = 0.80 (95% CI = 0.76-0.83)			
	PI-RADS v2, PSAD categories, and ADC categories			
**PI-RADS v2**				
4	2.25	0.008	1.23	4.11
5	6.58	<0.001	3.52	12.31
**PSAD categories**				
2	2.48	0.002	1.40	4.39
3	2.22	0.009	1.22	4.04
4	3.67	<0.001	2.21	6.10
**ADC categories**				
2	1.77	0.07	0.95	3.29
3	3.67	<0.001	2.07	6.52
4	8.78	<0.001	4.76	16.20

AUROC = 0.83 (95% CI = 0.79-0.86)				

PI-RADS v2, PSAD categories, ADC categories, age, and palpable nodule				

Age	1.03	0.031	1.00	1.06
Palpable Nodule	2.46	0.006	1.30	4.69
**PI-RADS v2**				
4	2.16	0.012	1.18	3.95
5	6.18	<0.001	3.30	11.58
**PSAD categories**				
2	2.46	0.003	1.36	4.46
3	2.21	0.01	1.20	4.07
4	3.75	<0.001	2.25	6.25
**ADC categories**				
2	1.76	0.07	0.95	3.23
3	3.46	<0.001	1.96	6.13
4	7.73	<0.001	4.18	14.3
**AUROC = 0.84 (95% CI = 0.81-0.87)**				

**P** = probability; **CI** = confidence interval; **PI-RADS v2** = Prostate Imaging Reporting and Data System, version 2; **AUROC** = area under the receiver-operating characteristic curve; **PSAD** = prostate-specific antigen density; **tz** = transition zone; **ADC** = apparent diffusion coefficient

Analyses stratified by location in the peripheral or transition zone showed did not significantly change the areas under the ROC curves, but, as may be expected, palpable nodule was not a predictor of clinically significant prostate cancer in the transition zone.

### Logistic Regression-Upgrade on Systematic Biopsy

**Figure 3:**
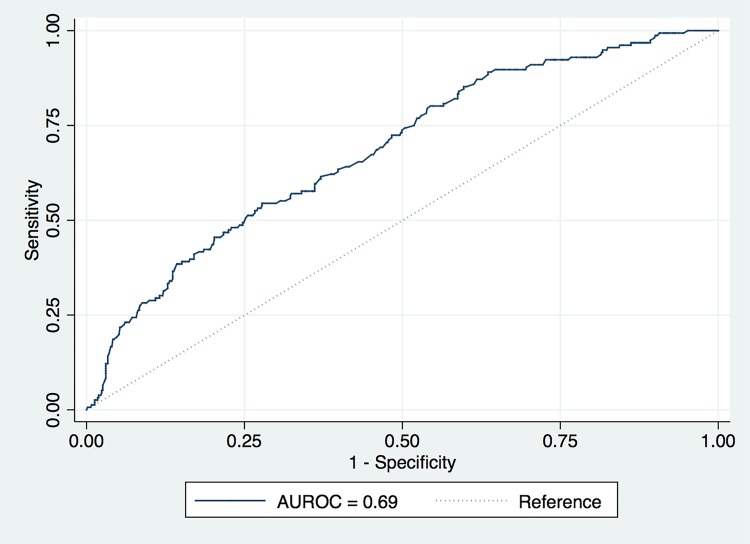
Prediction of upgrade on systematic biopsy, ROC curve. The area under the ROC curve of the model was 0.69, only marginally discriminating between men in whom systematic biopsy will and will not lead to upgrade to clinically significant prostate cancer.

Only categorical PSAD, and the diameter and number of lesions seen on mpMRI were identified by both forward and backward selection models for inclusion in the multivariate models. Additionally, the multivariate model also included PI-RADS v2 scores. Table-4 summarizes these results. The area under the ROC curve of the multivariate model was 69% (95% CI=64-73) (Figure-3).

**Table 4:** Upgrade of systematic biopsy – logistic regression analysis.

	Odds Ratio	P	95% CI	
**PI-RADS v2**				
4	1.20	0.41	0.78	1.84
5	0.48	0.02	0.26	0.88
**PSAD categories**				
2	2.39	0.003	1.35	4.21
3	2.39	0.008	1.25	4.54
4	2.48	0.003	1.37	4.47
Number of lesions on MRI	1.41	0.02	1.06	1.89
Lesion diameter on MRI	0.76	0.06	0.57	1.01
**AUROC = 0.69 (95% CI = 0.64-0.73)**				

**P** = probability; CI = confidence interval; **PI-RADS v2** = Prostate Imaging Reporting and Data System, version 2; **PSAD** = prostate-specific antigen density; **MRI** = magnetic resonance imaging; **AUROC** = area under the receiver-operating characteristic curve

## DISCUSSION

Our results show that PSAD and ADC values independently improve the PI-RADS v2 prediction of Gleason score ≥3+4 prostate cancer, and that utilizing their categorical versions is likely to have the same clinical impact. Also, adjustment of PSAD to the volume of the transition zone does not seem to provide additional information.

These results corroborate those of Jordan et al. (2), who found that ADC values below 800x10^−6^mm^2^/s improved the characterization PI-RADS v2 score 4 lesions in a population of men seen in a community clinic. More recently, Gaur et al. found that mean ADC values and normalized ADC helped to characterize lesions assigned a PI-RADS v2 score 4 or 5 (9). The authors did not categorize ADC values, but their ROC analysis suggested that a 1050x10^−6^mm^2^/s mean ADC value threshold increases the diagnostic accuracy of PI-RADS v2. This number is within our category of mildly low ADC values that were associated with at least doubling of the odds of clinically significant prostate cancer. Lin et al., however, did not identify an improvement in the diagnostic performance of PI-RADS v2 with the addition of ADC value measurements (10). It is difficult to explain this discrepancy, which is likely multifactorial. Potential reasons include variability of ADC values across imaging platforms and protocols (11), differences in the ADC value threshold used in each study, and inter-reader variability of PI-RADS v2 (12), which would impact the sensitivity and specificity of the method, and therefore the incremental usefulness of ADC values. Yet, because at any given institution these factors tend to be more or less constant, so does the range of ADC values that are measured. Thus, although the range may differ across imaging sites, ADC values can be easily obtained and should better characterize disease status of patients imaged at individual centers.

PSAD has been previously shown to improve the diagnosis and characterization of lesions seen on TRUS and mpMRI. Almost 30 years ago, Veneziano S. et al. showed that PSAD calculate using TRUS could identify men who had an elevated PSA due to BPH rather than cancer (13). Later, Catalona et al. showed that a PSAD ≤0.15 could be used to predict favorable pathology on prostatectomy (14). Similarly, PSAD can improve the accuracy of mpMRI; a PSAD ≥0.15 doubles the rate of clinically significant prostate cancer in men with PI-RADS v2 4 or 5 lesion (3). And Kotb et. al. suggest that re-biopsy is not necessary in men prior negative biopsy, PSAD <0.15, and low PI-RADS score (15). Our results show similar impact on the diagnosis of clinically significant prostate cancer. Although the area under the ROC curve of PI-RADS v2 and PSAD was statistically larger than the area under the ROC curve of PI-RADS v2 and ADC categorical, the difference between the two was not clinically relevant. While either approach can be used, the interpretation and clinical application of a categorical variable may be simpler and easier to understand.

PI-RADS v2 does not aim at identifying prostate cancer in areas without a visible lesion on mpMRI. Yet, it is known that around 5 to 20% of clinically significant prostate cancers are identified in such areas (6, 7). In our population, upgrade on systematic biopsy was slightly more likely to be seen in men with PSAD ≥0.15 and multiple small PI-RADS v2 3 or 4 lesions on MRI. The presence of a PI-RADS v2 5 lesion, though, made upgrade less likely, as these often already represent clinically significant prostate cancer. As size is one of the criteria to assign a PI-RADS v2 score 5 to a lesion, it is not surprising that small lesions are more likely than large ones to be associated with upgrade on systematic biopsy. It is important to note that multifocal clinically significant prostate cancer is not excluded by the presence of a PI-RADS v2 5 lesion. And multifocality may explain the association of multiple lesions on MRI and upgrade on systematic biopsy.

The PI-RADS guidelines asks for the sole assessment of imaging findings, but basic clinical data, e.g. total serum PSA, is routinely available to radiologists at the time of imaging interpretation. This data is utilized daily by urologists to assist with management decisions. Similarly, radiologists should not ignore other existing information, but incorporate these to practice to better serve our patients and colleagues. PI-RADS is a work in progress and, as new data becomes available, revised versions are expected to be released. Until large studies that investigate the impact of imaging on hard outcomes as death or metastases become available, the identification of clinically significant prostate cancer will continue to serve a surrogate marker. It is our hope that the results of this study help to develop a new version of PI-RADS, enhance the characterization of lesions visible on mpMRI, and improve the identification of men with clinically significant prostate cancer.

This study has limitations inherent to a retrospective, single institution research. The rate of clinically significant prostate cancer per PI-RADS v2 scores was lower than the average in the literature (16), suggesting a high sensitivity threshold of readers. This may reflect different experience of the various readers who interpreted the scans. Accordingly, the impact of ADC and PSAD of PI-RADS v2 may not be the same at other sites with different sensitivity and specificity profiles. This study was based on the review of medical charts, so images nor slides were re-analyzed. We made this option because we aimed at learning the value of using ADC and PSAD in daily practice, but the method is prone to errors in data collection. To minimize this problem, the authors who collected the data were trained, we used a standardized abstraction forms, and we had a quality and assurance process in place. Our standard of reference was not prostatectomy, but TRUS-MRI fusion biopsy, and therefore only samples of the gland were considered to determine our outcomes. We made this option because TRUS-MRI fusion biopsy is quickly becoming the standard of practice and to avoid selecting only men who underwent surgery, which would have inflated our sample with patients diagnosed with clinically significant prostate cancer.

## CONCLUSIONS

ADC values and PSAD help to characterize lesions that are assigned a PI-RADS v2 score 4 or 5 as clinically significant prostate cancer. The predictive value of categorized ADC values and PSAD are not markedly different from the continuous versions and can, therefore, be utilized in daily practice. Men with PSAD ≥0.15 and multiple small lesions assigned a PI-RADS v2 score 3 or 4 are marginally more likely to be upgraded on systematic biopsy.
